# “Do I feel safe here?” Organisational climate and mental health peer worker experience

**DOI:** 10.1186/s12913-024-11765-8

**Published:** 2024-10-18

**Authors:** Verity Reeves, Mark Loughhead, Matthew Anthony Halpin, Nicholas Procter

**Affiliations:** 1 University of South Australia, Clinical Health Sciences, Adelaide, South Australia Australia; 2 University of South Australia, Clinical Health Sciences, Adelaide, South Australia Australia; 3 University of South Australia, Clinical Health Sciences, Adelaide, South Australia Australia; 4 University of South Australia, Clinical Health Sciences, Adelaide, South Australia Australia; 5 The University of South Australia (Clinical Health Sciences) – GPO, Box 2471, 5001 Adelaide, South Australia Australia

**Keywords:** Peer support, Organisational culture, Organisational climate

## Abstract

**Background:**

In Australia, lived experience peer support workforces are expanding making it one of the fastest growing emerging disciplines in transdisciplinary mental health settings. This article provides insight for organisations on the contextual realities peer workers face, increases understanding of peer support services to improve service delivery environments and contributes to mental health systems reform. This study aimed to qualitatively explore peer support workers experience integrating into and working within transdisciplinary mental health service teams.

**Method:**

Semi-structured interviews were undertaken with 18 peer support workers currently working in mental health services in Australia. The research was a qualitative descriptive study design. All data collected were analysed utilising thematic analysis.

**Results:**

Peer workers found their experience in the workplace was influenced by their colleagues and the organisation’s understanding of the peer role. Factors relating to organisational culture and climate were a central theme throughout discussions noting that a negative climate was perceived as harmful to peer workers. Themes established through results include (1) the role of leadership, (2) attitudes and behaviours of colleagues, (3) provision of psychologically safe environments, (4) the organisations messaging and use of language and (5) organisational structures and policy.

**Conclusion:**

This study contributes to evidence for the impact of organisational culture on integrating and supporting peer support workers in mental health service delivery. This study provides insights into peer worker experiences integrating into transdisciplinary teams, confirming findings established in previous studies, highlighting a lack of movement or change in workplace culture to support peer worker integration into mental healthcare settings.

## Background

Mental health service reform has seen an increase in participation and employment of service users within transdisciplinary mental health service delivery. The inclusion of service users in the design and delivery of mental health services is a significant component of the recovery approach and a key indicator in The Fifth National Mental Health and Suicide Prevention Plan [1, 2]. While there are many roles that service users and carers undertake in service reform, peer workers are people who utilise their lived experience expertise and peer support skills to help others on their recovery journey [3–5]. For this study, the term ‘service user’ will be utilised when referring to a person who actively utilises and engages in mental health service use.

The recovery approach highlights that people experiencing mental health conditions can live a meaningful and purposeful life; a life they have chosen, despite the presence of symptoms [6, 7]. Principles of recovery practice emphasise having autonomy and informed choice, activating empowerment and hope for the future utilising an individual’s unique strengths and characteristics on their recovery journey [8]. Previous research has found peer workers can inspire hope and empowerment for help seeking behaviour, role model recovery processes and reduce instances of crisis resulting in hospitalisation [9–12]. Peer workers offer authentic connection, often helping service users to navigate services and relationships and improve quality of care [13, 14].

Despite identified benefits of the active participation of peer workers in service delivery, poor working conditions and remuneration raise concerns about peer workers ability to have meaningful impact within the system [15–17]. Further, recent reviews of evidence identify organisational culture, role identity and a lack of understanding of the role as key barriers to the integration of peer support in services [18–20].

The history of inquiries into the state of Australian mental health services present a flawed system of disconnected services, unprepared to accommodate or validate a culture of shared decision making or sufficiently cope with current and growing demand [21–25]. Mental health reform in Australia has seen the development of consecutive mental health plans which highlight the need for cultural change, promote consumer rights and improve cultural and psychological safety throughout mental health service settings [7]. Currently in Australia, lived experience peer support workforces are one of the fastest growing disciplines with an annual growth rate of 15.9% from 2015 to 2020 [26]. This is supported and endorsed through national government scholarship programs offering people with lived experience the opportunity to complete a Certificate IV in Mental Health Peer Work. This is in addition to an $8.5 million dollar investment in lived experience peak bodies and ongoing development of lived experience workforce guidelines and mental health stigma reduction strategies as part of the Australian Government’s commitment to developing and growing mental health workforces [3, 27–29].

Such significant and ongoing investment in lived experience workforces seeks to improve visibility and access to peer services in mental health organisations, while also acknowledging the need to shift current service culture and delivery to meet service user needs. Organisational culture refers to the shared aspects of an organisation including values, norms and shared behaviours that impact a workers experience in the workplace [30]. A positive organisational culture is essential to establishing productive working environments and has been linked with improving the quality of services delivered and worker performance [30–32]. An organisations climate is distinguished from cultural norms and can be defined as an employee’s perception of the psychological impact a workplace has on their functioning and well-being [33, 34]. These concepts are important to support inclusion of peer workers in mental health organisations, achieving systems reform, and promoting recovery aligned care models [35]. Despite acknowledgment of appropriate organisational culture and climate being essential for integrating peer and lived experience workforces, significant challenges remain to ensure appropriate valuing of lived experience knowledge and expertise at the point of care.

As the peer workforce grows and workers are introduced into existing transdisciplinary working teams, influencing mental health service delivery, it is critical to understand barriers and enablers for worker experiences to promote successful role integration, acceptance, and sustainability. It is evident from literature that organisational climates vary across global contexts [18, 36]. There are many variables shaping the successful integration of peer workers including climate, recovery culture/biomedical approach, stigma, deficit thinking and a lack of valuing of lived experience expertise [3, 18, 19]. Against this background, this study sought to explore an Australian sample of peer workers’ experiences and the perceived influence of an employing organisation’s culture and climate on their integration into transdisciplinary mental healthcare teams.

## Methods

This research utilised a qualitative descriptive study design and aimed to explore peer workers experiences working in mental health organisations. In healthcare research, a qualitative descriptive design allows for a straightforward description of a person’s perception or experience, appropriate for this study as it recognises the subjective nature of personal experience and presents findings relevant to the intended research questions [37]. This research sought to explore these experiences to enable greater understanding of the impact an organisation can have on peer worker integration and support sustainability of the peer support role.

### Participant recruitment

Snowball and purposive sampling techniques were utilised and recruited 18 peer support workers currently working within mental health organisations across four Australian states. See Table 1 for participant demographics. All participants were currently employed and working in a peer worker or carer peer worker role within mental health organisations in Australia. Participants experience ranged from 3 months to 20 years. Each participant was asked to reflect on and share recent experiences to ensure current industry relevance for this research. Recruitment materials were distributed electronically and advertised via website and social media platforms. Participants were provided detailed information sheets about the project. Signed consent was received from participants prior to interview.

**Table 1:** Participant demographics

**Gender**	**Number (n)**
Male	6
Female	12
Non-binary	0
**Organisation type**	
Government Organisation	6
Non-Government Organisation (NGO)	12
Lived experience led organisation	0
**Identify as having a disability**	
Yes	6
No	12
**Identify with ethnic or cultural group**	
Yes	7
No	11
**Peer worker**	17
**Carer Peer worker**	1

### Interviews

All semi-structured interviews were conducted by the first author (VR) via telephone or videoconferencing over a 6-month period. Semi-structured interviews lasted between 20 and 80 min (average of 38 min). All interviews were recorded, de-identified and professionally transcribed verbatim. Additional data and results gathered from this interview schedule is published elsewhere [38].

### Ethics

Ethical approval was granted for this research through the University of South Australia’s Human Research Ethics Committee (ID: 203551).

### Analysis

Interview transcripts were analysed utilising a thematic analysis, following steps outlined in Braun and Clarke [39]. Qualitative content analysis was used to group and draw themes from the raw data [40]. This approach allowed for an in-depth analysis and exploration of each participant’s experiences and perspectives. Data were individually coded and similar codes from each transcript were grouped to establish preliminary themes using NVivo 2020 v1.6 software. Preliminary themes were discussed by co-authors and refined into a coherent narrative of peer workers experiences.

The author’s brought differing knowledge backgrounds to the analysis including lived experience, social sciences, and mental health nursing. Yardley’s [41] principles for evaluating the quality and validity of the data were used. *Sensitivity to context* was encouraged by gaining a robust and in-depth understanding of current relevant literature and industry context prior to the collection and analysis of the data. *Commitment and rigour* were enhanced by authors (VR and ML) coding the first 5 transcripts, bringing together social science and lived experience researcher perspectives. All authors were involved in the process of theme development utilising knowledge of service contexts, lived experience leadership and peer support practice to inform analysis. Data saturation was indicated (by VR) after 18 interviews when no new information or patterns were emerging from the data and interview questions were met with consistent responses by participants within both government and non-government organisations. Utilising a well-established approach to the data collection and analysis, Braun and Clarke [39] and reporting on key methods contributed to the study’s *transparency*. *Coherence* was established through the choice of thematic analysis as a theoretical framework which was appropriate for the exploratory aim of this research. Authors prioritised reflexivity to gain insight into how researcher motivations and experience may influence the interpretation of the data and subsequent analysis. This practice occurred through regular discussion between authors on their positionality to the topic and their own perspectives.

## Results

*“My decision came to resigning from that position because the environment was too toxic” P9*.

Five themes emerged from analysis of participant data which consistently highlighted the impact of an organisations culture and climate on a person’s experience integrating into a new role, see Table 2 below. Each theme is illustrated throughout results by participant quotes.

**Table 2:** Distribution of themes across participants

Theme	Number of participants who made reference	Total number of references to theme
Attitudes and behaviours of colleagues	16	50
Organisational messaging and use of recovery language	15	67
Psychologically safe environments	11	32
Role of leadership	15	41
Organisations structures and policies	14	44

### Attitudes and behaviours of colleagues

When discussing the challenges faced throughout their role, 16 of the 18 participants reported stigma and negative attitudes from colleagues as a key barrier to their acceptance and integration into transdisciplinary teams.

*“Attitudes about people with lived experience have been that oh maybe they need a safe space*,* or they’re more likely to be sick*,* or their emotional resilience is weaker than somebody else’s so they wouldn’t be able to cope” P1*.

*“They’re thinking that*,* oh*,* what’s-a-name is living with a psychological distress; therefore what’s-a-name is not capable for the role.” P3*.

Participants noted being ostracised by team members, not being taken seriously, feeling tokenistic; consistently needing to justify their position to gain acceptance within the team.

*“Having to constantly prove your abilities and prove the skillsets and so many different aspects of who you are*,* it’s really quite challenging” P9*.

*“Spoken over the top in meetings*,* or ignored*,* or not being taken seriously*,* not being listened to*,* and people’s egos – they think they’re better than you… I was made to feel like an outsider*,* isolated… I couldn’t really talk to many people” P10*.

Conversely, several participants reported positive experiences and perceived greater acceptance when colleagues understood lived experience, leading to greater valuing of the role and knowledge peer workers bring. Participants suggested greater education on the role and value of peer work to be essential in reducing instances of stigma and facilitating greater acceptance of peer roles in transdisciplinary teams.

## Organisational messaging and recovery language

Participants noted consistent messaging utilising strong recovery language from the organisation on the value and utility of peer support favourably impacted workers acceptance in teams.

*“As they understood me and my job*,* then they understood*,* and they’d come and seek me out to help them.” P10*.

Use of strong recovery language and terminology played a crucial role in promoting acceptance and integration, particularly during team meetings, in written case notes and organisational value statements.

*“Keep the recovery framework strong and the language strong on the ward. Look at it from a strength-based perspective*,*” P9*.

Where poor recovery focused language often proved to be a barrier to peer workers feeling safe and accepted as part of the team.

*“I just don’t want to go back into that environment where I’m hearing really invalidating comments*,* very judgmental comments about the young people using our service.” P17*.

*“Really have to be very conscious of the terminology they use*,* the way they identify people with mental illness” P5*.

## Psychologically safe environments

*“Someone had said to me*,* “Don’t share your story here because it’s not safe.”” P9*.

Provision of psychologically safe environments for peer workers underlies their self-perception of being valued. Creating a space where a peer worker feels comfortable to share aspects of their story and utilise this experience to support others is crucial to effectively executing the peer role and promoting worker sustainability.

*“We don’t feel safe to speak or to bring our lived experience because of the reactions we get from the team. And it really should be a space where we can respectfully challenge ideas” P17*.

*“It’s really hard to work amongst that environment because it was – how can I say it? I was questioning – my inner narrative was questioning myself*,* “Do I feel safe here? Do I actually feel safe here?”” P9*.

Participants also highlighted working within an organisational climate that was not conducive to supporting lived experience expertise as potentially causing harm or being re-traumatising.

*“The other thing that was quite distressing for me is the other peer workers were not feeling valued either… I will use this word as well*,* we were being psychologically harmed in the workplace. So really strong words and I put up with a lot” P17*.

Conversely, participants who were part of a supportive culture felt valued and respected and enjoyed their role and contribution to the team, highlighting the impact positive and supportive culture has on a peer workers integration into mental health organisations.

*“I’m in a team*,* where I’m heard and respected and my opinion has some weight*,* so that’s one of the things that I definitely enjoy” P15*.

### Role of leadership

The impact a leadership team has on an organisations climate and the experience of the worker was highlighted by 15 of 18 participants within this study.

*I think leadership is vitally important and crucial. Leadership has the ability to implement direction*,* set culture*,* uphold values*,* set an example - there’s so much that a leader can do. P7*.

Participants suggested organisational leaders have the capacity to promote and drive improved workplace understanding of the peer role, provide opportunities for integration and promote buy-in for a whole-of-organisation approach to recovery orientated care.

***“****Provide those opportunities to facilitate space for peer workers and lived experience workers to share their knowledge and to share their experience to help other people understand the value of what a lived experience practitioner can bring to a client*,* to people that you support.” P11*.

A leaders’ ability to provide support through professional supervision, lived experience specific training and education on the peer role was considered to facilitate overall understanding and acceptance among non-peer colleagues highlighting the need for consistency from leadership at all levels of the organisation to support and value expertise brought by peer workers.

### Systems and policy structure

How an organisation structures their workforce to include peer workers and the development of policy to support a recovery framework and value lived experience expertise was a key influence over a peer workers experience. Specifically, the inclusion of peer support as a regularly offered service and the promotion of peer services throughout the organisation.

*“Being able to highlight the particular skills and experiences that each peer worker has*,* is going to be vital in terms of effectively engaging in that new workplace” P2*.

Inclusion of peer workers in the development of policy was suggested as a way for peer workers to facilitate greater inclusion within mental health organisations.

*“Before you design a policy*,* maybe during – while you design the policy just look*,* hey*,* come down to us and ask us for advice.” P3*.

## Discussion

The intention of mental health services globally is to provide a safe environment for people to go to in times of crisis, to provide pathways to care and support and empower life choices. To ensure services can meet these requirements, organisations have an obligation to cultivate workplace settings where peer workers are able to utilise their expertise to provide services for service users. Through exploration of peer workers experiences working within mental health organisations, this study finds organisational culture and climate as key influences over the worker’s perception of positive and negative experiences within the workplace. These have been identified via five themes: (i) attitudes and behaviours of staff, (ii) the messaging and language utilised in workspaces, (iii) the provision of psychologically safe environments, (iv) organisational structures and policy and (v) the role of leadership teams.

The attitudes and behaviours of workplace colleagues were recognised as a major influence over a peer workers integration and feeling of acceptance, whether positive or negative, within a team or organisation. Negative experiences dominated participant responses and was, in part, attributed to a lack of understanding of the peer worker role and pre-existing stigmatising attitudes towards people with mental health diagnoses. It is very concerning that stigma and devaluing attitudes persist, given that these experiences have been reported in many peer work studies [42–45]. Positive experiences documented by participants tended to be prominent in environments where colleagues understood and welcomed peer expertise as a valued contribution and an additional tool to assist people on their recovery journey. It reinforces the need for continual development of lived experience workforce frameworks and education to reduce negative attitudes, facilitate open conversations within teams and build positive relationships between co-workers [46, 47].

An organisation’s culture is central in shaping the attitudes and behaviours of its staff, however shifting negative or detrimental attitudes and behaviours within an existing workplace can be challenging and difficult to embed [48]. Themes established in this research found leadership support and consistent organisational messaging to influence a peer workers experience integrating into transdisciplinary teams. This is consistent with theoretical frameworks for organisational culture change, with leadership support and clear communication identified as core components in preparing, managing and reinforcing change to facilitate cultural shift [49, 50]. Leadership support, including partnership with lived experience leaders, and consistent clear messaging is supported through literature as successfully supporting integration and sustainability of the peer support role [18, 47, 51].

Closely tied to leadership and organisational messaging is the use of inclusive and recovery focused language within workplaces. Peer worker participants in this study were acutely aware of the impact of unsupportive language used by staff, as a feature of negative culture impacting on service users as well as their own sense of how recovery is valued by the service. Words and language form part of an inner narrative and discourse utilised to create and understand something and, ultimately, influences how a person perceives their objective reality [52]. Traditional biomedical models of mental health care, in conjunction with illness classification manuals such as the DSM-5 and ICD-11, have normalised and encouraged the use of clinical language in treatment of mental illness. As reported in this study, language and use of clinical terminology or labelling was considered ostracising by study participants or incongruent with how a person perceived themselves and their recovery journey. While services have made significant changes toward adoption of biopsychosocial models of care, use of language and persistent negative attitudes from colleagues have proved ongoing challenges for peer workers [52, 53]. It must be acknowledged that while some may find use of diagnostic labelling or classification systems stigmatizing, they are essential to guide treatment approaches. It is crucial then that allied health practitioners recognise and adopt person-centred approach to care and discuss individual preferences in regard to language and approach to treatment options. Glover [54] reminds us that a paradigm shift towards recovery requires deep questioning of assumptions and language prevalent within culture and care models so that shifts in language occur from service centred to person centred ways of recognising distress, agency, decision making power and responsibility. Accompanying this shift is the development of new designated lived experience positions which specifically facilitate learning, hope, connectedness and empowerment and contribute to developing inclusive and accepting workplace cultures [55–57].

As highlighted in this study, a psychologically safe environment is one in which an individual feels safe to provide feedback, voice ideas and take risks contributing to overall organisational learning [58, 59]. Crucial to peer work is the sharing of lived experience which can be challenging in environments where workers feel unsafe or unvalued for the expertise they bring. While lack of psychologically safe workplaces was noted as a persistent barrier for participants when integrating into transdisciplinary teams, evidence suggests the provision of a psychologically safe workspace promotes greater work participation, social inclusion, and increased job satisfaction [59, 60]. Providing psychological safety is a key determinant of safe and effective mental health service provision, particularly in recovery-orientated and trauma-informed approaches which requires collaborative input from all transdisciplinary team members [60, 61]. The findings in this data contribute to a growing literature base investigating the current state of psychologically safe workplaces within mental health organisations, indicating significant work is required.

## Conclusions

Overall, previous research and the findings of this study suggest there are significant challenges faced by mental health organisations integrating and promoting sustainability of peer support roles within transdisciplinary service teams. This study is consistent with findings previously reported in the literature concerning the persistent challenges peer services encounter establishing their place within mental health service contexts. Findings consider the experiences of peer workers in the context of organisational culture and climate and highlights that encouraging effective organisational change requires in-depth consideration and conceptualisation to establish meaningful change.

### Limitations

This research may be limited due to smaller sample size (*n* = 18) and future research may benefit from including a larger sample. The findings are limited to peer worker and carer peer workers and may not be generalizable to all other lived experience designated roles. As the sample included both government and non-government services, there are limits to the generalisability of findings to each service area. The use of purposive and snowball sampling to recruit participants can present potential for selection bias due to self-nomination in the study which may be influenced by interviewees with strong views on actions to address challenges faced. The authors note that findings reported in this article are not the sole outcomes of the study with an additional publication reporting of outcomes pertaining to peer support work and organisational practices [38].

## Data Availability

The data that support the findings of this study are available from the corresponding author, VR, upon reasonable request.
